# An accurate irrigation volume prediction method based on an optimized LSTM model

**DOI:** 10.7717/peerj-cs.2112

**Published:** 2024-06-13

**Authors:** Hui Yan, Fahuan Xie, Duo Long, Yunxin Long, Ping Yu, Hanlin Chen

**Affiliations:** 1 School of Information Engineering, Suqian University, Suqian, Jiangsu, China; 2 Jilin Province S&T Innovation Center for Physical Simulation and Security of Water Resources and Electric Power Engineering, Changchun Institute of Technology, Changchun, Jilin, China; 3 School of Management, Suqian University, Suqian, Jiangsu, China; 4 College of Traditional Chinese Medicine, Changchun University of Chinese Medicine, Changchun, Jilin, China

**Keywords:** Accurate irrigation volume prediction, Attention mechanism, Agricultural production, Temporal information, Spatial features, BiLSTM-CNN-Attention

## Abstract

Precise prediction of irrigation volumes is crucial in modern agriculture. This study proposes an optimized long short-term memory (LSTM) model-based irrigation prediction method that combines bidirectional LSTM networks. The model provides farmers with more precise irrigation management decisions, facilitating optimal utilization of water resources and effective crop production management. This proposed model aims to fully exploit spatio-temporal features and sequence dependencies to enhance prediction accuracy and reliability. We aim to fully leverage crop irrigation volumes’ spatio-temporal features and sequence dependencies to improve prediction accuracy and reliability. First, this study adopts a bidirectional LSTM (BiLSTM) model to simulate the temporal features of irrigation volumes and learn the sequential dependencies of crop growth data from historical records. Then, this study passes the irrigation volume data through a convolutional neural network (CNN) model to extract spatial features and capture correlations among various features such as temperature, precipitation, and wind speed. Our prediction performance significantly improved after incorporating an attention mechanism that involves weighting features and enhancing focus on crucial aspects. The proposed BiLSTM-CNN-Attention approach is used to predict irrigation volume for spring corn in significant irrigation areas in Jilin Province, China. The results demonstrate that the proposed method surpasses recurrent neural network (RNN), CNN, LSTM, BiLSTM, and BiLSTM-CNN methods in terms of mean square error (MSE), root mean square error (RMSE), mean absolute error (MAE) (0.000004, 0.005968, 0.004599), and R^2^ (0.9749), making a superior solution for predicting the volume of crop irrigation.

## Introduction

According to a recent study prediction from global water resources data, two-thirds of the world’s population will face water shortages in the coming decades, severely affecting livelihoods and agriculture (Rijsberman, 2006). Unfortunately, water scarcity significantly constrains agricultural water resources and expansion of irrigated areas in numerous regions (Li et al., 2021; Wang et al., 2014). The lack of water for irrigation has become a significant issue as crop acreage and agricultural water demand rise. Prioritizing the development of innovative irrigation water conservation technology is crucial to meet the demands of high-standard farmland. This technology can significantly reduce water usage while maintaining the high quality of the farmland (Li, 2023). Therefore, utilizing artificial intelligence (AI) techniques to improve irrigation strategies and predict irrigation water volumes is crucial for enhancing irrigation water efficiency and achieving maximum crop yields.

As the world continues to witness remarkable advancements in AI and technological innovation, intelligent irrigation systems have emerged as promising methods for irrigation. Studies have used Penman’s formula and neural network algorithmic models to predict crop water requirements, enabling farmers to manage irrigation more effectively. In agricultural irrigation, studies have also found that crop water demand is affected by internal and external factors (Jia & Liu, 2019). Internal factors include differences in water requirements between different crop types and their different growth stages. In contrast, external factors include environmental factors such as temperature, humidity, sunlight, wind speed, transpiration evaporation, and irrigation water (Zhang, Xu & Song, 2021). The variation in water requirements for crops at different growth stages is ignored in the irrigation process. Ignoring this can reduce crop yield and quality, as traditional empirical irrigation with large volumes of water will harm crop yield and quality (Xu, 2019). Therefore, we must consider meteorological factors, soil moisture conditions, and crop characteristics to achieve precision irrigation. This study proposes building a database of water demand for different crop types and growth stages to accurately compare and analyze water demand during irrigation and achieve on-demand irrigation. Therefore, the study investigates irrigation strategies at actual sites.

The proposed BiLSTM-CNN-Attention-based model aims to achieve accurate crop irrigation prediction by combining several powerful deep-learning techniques. The model is trained and tested on irrigation data from 2000 to 2022 for central irrigation districts in Jilin Province, China. The model uses a combination of bidirectional long and short-term memory networks (BiLSTM), convolutional neural networks (CNN), and attention mechanisms. Specifically, CNN extracts critical features from input irrigation data, while BiLSTM can capture complex dependencies among stock time series data. Additionally, the attention mechanism is introduced to enhance the model performance by accurately capturing the impact of past feature states on the irrigation volume in time series data. Integrating these components has led to significant improvement in the accuracy of crop irrigation prediction. This work makes several contributions toward improving our understanding of crop irrigation prediction and its practical applications in agriculture. This study offers several main contributions:

First, we employ the Aqua Crop model to optimize irrigation strategies for crop characteristics and environmental factors based on meteorological data, crop data, and Penman’s formula. Calculating crop demand irrigation in a given area can improve irrigation effectiveness and water usage on farmland.

Second, irrigation water demand is a complex and dynamic process affected by various factors such as temperature, rainfall, and wind speed. This study proposes an innovative deep-learning approach based on the BiLSTM-CNN-Attention model for irrigation volume prediction. This model utilizes BiLSTM and CNN layers to maintain sequence information and incorporates the attention mechanism to understand the impact of historical feature states on irrigation volume. The result offers a more accurate prediction model for irrigation volume.

Third, this study has taken a specific approach to optimizing the sequence coding capabilities of BiLSTM. The loss of sequence information limits the efficacy of the BiLSTM layer; however, this can be reduced by placing the BiLSTM layer ahead of the CNN layer in the processing pipeline. Prior research has deliberately chosen to utilize the BiLSTM layer before a CNN layer. This decision was based on the concern that applying a CNN layer might cause sequence information to be lost, which could impede the effectiveness of the subsequent BiLSTM layer to maximize the potential of BiLSTM’s sequence coding capability. Additionally, introducing the attention layer helps clarify the degree of influence of historical feature states on the irrigation volume, further improving the prediction accuracy.

Fourth, this study introduces a new approach to improving a model’s stability and generalization ability through residual networks. By adding a residual network between the 1-layer convolution and the 3-layer convolution, the problem of saturation and fading in the accuracy of the training set can be alleviated, and the gradient explosion can be improved. This approach appears to have positive implications for improving the model stability and generalization.

Fifth, we compared five machine learning methods for predicting crop irrigation volume through experiments. The results show that the BiLSTM-CNN-Attention method outperformed other methods regarding prediction accuracy and effectiveness. These findings validate the superiority of the BiLSTM-CNN-Attention approach for crop irrigation volume prediction.

## Related Work

Numerous studies have dedicated their efforts to the precise prediction of crop irrigation levels by categorizing methods used for predicting crop irrigation levels into two: traditional statistical techniques, including regression analysis, quota method, and grey prediction method (Guo et al., 2017; Zhang & Zhang, 2013; Liu et al., 2021; Ohana-Levi et al., 2021) and machine learning, such as support vector machines, random forests, and artificial neural networks (Lian et al., 2023; Ohana-Levi et al., 2021, and Jianxing, 2021; Rahimi Khoob, 2008). Traditional mathematical models often exhibit limited accuracy in predicting crop irrigation amounts due to the fluctuating and distinctly nonlinear nature of factors influencing crop irrigation levels. This limited accuracy makes deep learning methods emerge as practical tools for describing nonlinear feature data, garnering widespread attention in crop irrigation level prediction.

Yufeng (2022) proposed an agricultural water-saving irrigation prediction algorithm that used a genetic algorithm (GA) to optimize the backpropagation (BP) neural network. The conclusion drawn from their study is impressive because it shows that the application of the GA-BP neural network agricultural water-saving irrigation prediction algorithm achieves high accuracy in predicting crop water requirements. This algorithm proves effective in achieving water-saving irrigation objectives and exhibits strong adaptability. Liu et al. (2021) employed a deep neural network (DNN) artificial learning model to construct an irrigation volume prediction model for bell peppers. This model accurately forecasts the irrigation needs of bell pepper crops and significantly reduces irrigation water usage. Zhou et al. (2018) utilized the combination of long short-term memory (LSTM) and wavelet decomposition to predict crop irrigation water requirements, which proved to be more accurate than the traditional autoregressive integrated moving average (ARIMA) models and BP neural networks. Akay et al. (2018) combined LSTM neural networks with Elman recurrent networks to predict crop irrigation water requirements. The results indicate that this method is more accurate than traditional support vector regression models and BP neural networks. Docheshmeh Gorgij et al. (2023) investigated the capability of LSTM method in forecasting the water quality for irrigation purposes *via* sodium absorption ratio (SAR) spatiotemporal modeling. The assessment results acclaim the usage of LSTM in forecasting irrigation water quality. The results indicated that LSTM and Bi-LSTM models exhibit higher accuracy in reference evapotranspiration prediction than traditional support vector regression models and BP neural network models.

Deep learning techniques have successfully addressed the challenges of crop irrigation volume prediction. However, these models still face information loss and accuracy challenges as the task complexity increases. We have introduced attention mechanisms to address these issues, enabling the model to focus on critical factors for enhancing adaptability dynamically. Attention mechanisms have become widespread across various fields. The research community has made significant efforts to develop crop-irrigation volume prediction models, adopting attention mechanisms to selectively focus on relevant input indicators, thereby improving prediction accuracy. Wang et al. (2022) proposed a BP neural network model optimized with a GA and an AdAttention algorithm for agricultural irrigation volume prediction. This model was applied to seven typical irrigation zones in the Longzhong region of the Yellow River basin. The model utilized meteorological data and actual measured irrigation data for corn, and they exhibited favorable prediction results. Zhang & Zhang (2013) employed a dual-stage attention mechanism-based approach to address the issue of feature data correlation in soil moisture prediction. The study introduced feature and time attention mechanisms building the DA-LSTM-soil model by constructing an encoder–decoder structure using LSTM networks. The results demonstrated that the DA-LSTM-soil model had higher accuracy, outperforming in metrics such as mean square error (MSE), root mean square error (RMSE), mean absolute error (MAE), and R^2^.

Researchers commonly employ machine learning methods to enhance accuracy in irrigation volume prediction. However, traditional neural network models pose several challenges when addressing irrigation volume prediction problems. First, the long-term dependency in the time series data of crop growth requires CNN models to capture this dependency and produce more accurate prediction results. Second, traditional neural network models exhibit limited local perception capabilities, making it challenging to effectively utilize local feature information in sequential data and susceptible to the influence of local noise. Moreover, traditional neural network models lack a mechanism for adaptively adjusting the importance of different sequence parts, specifically an attention mechanism, which limits the model’s ability to focus on critical information.

This study proposes the BiLSTM-CNN-Attention model to address these challenges, integrating BiLSTM, CNN, and an attention mechanism. This model aims to overcome the challenges above by enhancing irrigation volume prediction accuracy. The model is designed to tackle time series data challenges and extract local features using attention. This model has also shown promising results in irrigation predictions. However, further research and empirical analysis are required to validate the model’s performance across different datasets and environments.

### Construction of the model

#### Construction of the irrigation water demand model

**Figure 1:**
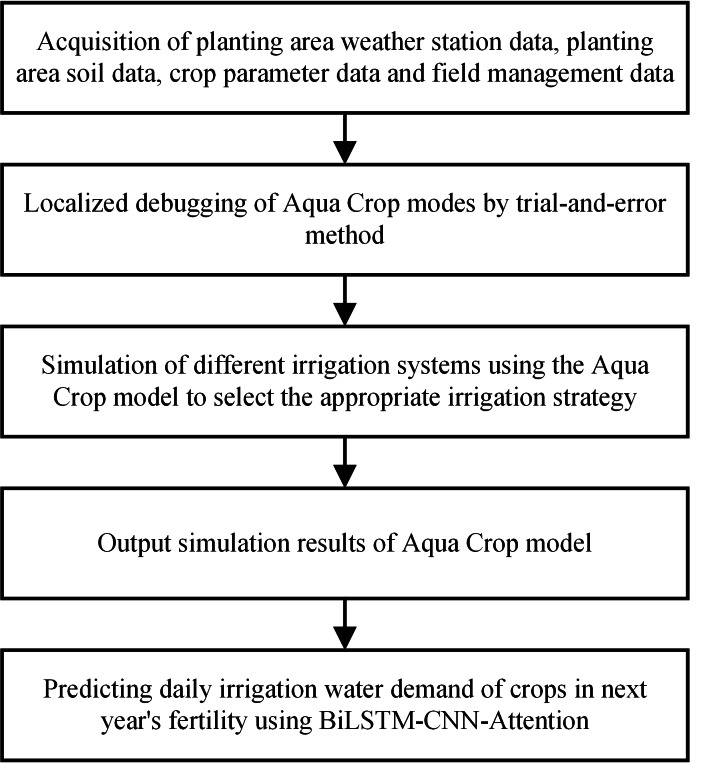
Location map of the research site.

This article has used the AquaCrop model to construct an optimal irrigation water demand model. Based on principles of crop physiology and soil water balance, the model provides an accurate estimate of crop irrigation requirements by combining factors such as meteorological data, soil properties, and crop parameters. We have combined the AquaCrop model with the BiLSTM-CNN-Attention algorithm to determine the optimal irrigation strategy and accurately predict the water requirements for crop irrigation, as shown in Fig. 1.

The detailed process for performing the proof has been outlined as follows:

The first step involves acquiring various meteorological station data, soil data, crop parameter data, and field management data for the planting area. The second step involves local debugging of the AquaCrop model through a trial-and-error method. Third, the model simulates different irrigation systems using the AquaCrop model to select an appropriate irrigation strategy. Then, the output of AquaCrop model simulation results is obtained. Finally, the BiLSTM-CNN-Attention model was used to predict the daily irrigation water requirement of the crop for the following year’s reproductive period.

This study combines crop models, machine learning methods, and existing meteorological stations and crop management data. We select suitable irrigation strategies based on the water supply and demand conditions in different areas and the water demand characteristics of crop growth.

Based on Chen et al. (2019), Zhang & Zhang (2013), Kuschel-Otárola et al. (2020), and Aemro, Mekete & Hailu (2022) Mathias models, we developed five irrigation strategies, namely rainfed, 80 mm irrigation, 60 mm irrigation, 45 mm irrigation, and optimal irrigation. Table 1 shows our specific irrigation strategies. Using the results of the AquaCrop model simulations, we select the best irrigation strategy based on the water requirements of each crop growth cycle.

**Table 1:** Irrigation strategies.

Irrigation strategies	Acronyms	Standard
Rain irrigation	RF	The crop’s water needs can only be met by rainfall
80 mm irrigation	80 CI	When soil water content (SWC) reaches a threshold value, crops are irrigated with 80 mm of water.
60 mm irrigation	60 CI	When soil water content (SWC) reaches a threshold value, the crop is irrigated with 60 mm of water.
30 mm irrigation	45 CI	When soil water content (SWC) reaches a threshold value, the crop is irrigated with 45 mm of water.
Optimal	TBI	Irrigate the crop according to the amount of water required. Irrigation is carried out according
Irrigation		To the date and depth calculated.

The Penman–Monteith method recommended by the Food and Agriculture Organization of the United Nations (FAO) calculates crop water requirements and irrigation. This method calculates ET0, as shown in Eq. (1):

(1) \begin{eqnarray*}E{T}_{o}= \frac{0.408\Delta \left( {R}_{n}-G \right) +\gamma \frac{900}{T+273} {U}_{2} \left( {e}_{S}-{e}_{a} \right) }{\Delta +\gamma \left( 1+0.34{U}_{2} \right) } \end{eqnarray*}

where *ET*_0_ is the reference crop evapotranspiration and *mm*/*dR*_*n*_ is the net canopy surface radiation .*MJ*/(*m*^2^⋅*d*)*G* is the soil heat flux ; G = 0 .32*MJ*/(*m*^2^⋅*d*)*γ* is the thermometer constant; *T* is the mean air temperature; ^0^*CU*_2_ is the wind speed at 2.0 m above ground level; and *e*_*s*_ is the saturated water vapor pressure of air. *KP*_*a*_*e*_*a*_ is the actual water vapor pressure of air, and *KP*_*a*_Δ isthe slope of the soaking water vapor pressure–temperature relationship curve.

We determined the fixed irrigation schedules and crop water requirements using CROPW version 8.0, based on FAO-56 (Bodner, Loiskandl & Kaul, 2007; Kassaye et al., 2020). The reference irrigation treatment (*K*_*c*_) had a crop factor of 100%. For the spring maize crop, the crop coefficients used in the reference irrigation treatment (*K*_*c*_) were 100%, based on the nutrient growth stages, which were 0.42 at the seedling stage, 0.753 at the growing stage, 1.288 at the flowering set, and 0.769 at maturity. For the soybean crop, the growth coefficients were 0.40 at the seedling stage, 0.64 at the growing stage, 1.14 at the flowering set, and 0.68 at maturity. We obtained 0.68. *K*_*c*_ for each growth stage from the literature (Li et al., 2020; Wang, 2020), and crop evapotranspiration (*ET*_*c*_, mm) was determined from Eq. (2):

(2) \begin{eqnarray*}E{T}_{c}=E{T}_{0}\ast {K}_{c}\end{eqnarray*}

where *ET*_0_ is the reference evapotranspiration in mm. Since the model is based on evapotranspiration, the net irrigation water requirement (*NIR*) can be quantified by subtracting the adequate rainfall for the experimental season (*P*), described by Eq. (3):

(3) \begin{eqnarray*}\mathrm{NIR}=\mathrm{E}{\mathrm{T}}_{\mathrm{C}}-\mathrm{P}.\end{eqnarray*}

#### Grey correlation analysis

Crop water requirements depend on several factors, including climate, soil conditions, and physiological properties. Temperature, humidity, wind speed, and hours of sunshine are critical factors influencing crop water requirements. We selected six parameters affecting crop water requirements and trained a neural network model to predict irrigation requirements. To determine the correlation between each element and the crop water requirement varies, we used the grey correlation analysis between the parameters affecting the crop water requirement and the prediction target.

The grey correlation algorithm (GCA) has a multi-step process to analyze crop impact factors. The first step is to identify the parent series and subsequences related to analyzing the crop impact factors. In the sample column, the parent sequence is the crop water requirement. In contrast, daily maximum temperature (Tmax), daily minimum temperature (Tmin), average temperature (Tmean), average wind speed, sunshine hours, and relative humidity are subsequences affecting the parent sequence. These factors are imported into the Python seaborn library for training, and the correlations between individual feature values are calculated. The relevant factors are selected based on the correlations as input factors for predicting the BiLSTM-CNN-Attention model.

The impact factors were analyzed in three steps using grey correlation.

Step 1: We determine the parent series as crop water requirement Y, $\text{where}Y= \left\{ Y \left( K \right) \setminus \right. $K = 1 $ \left. ,2,\ldots ,n \right\} .{T}_{\text{mean}},T{{T}_{min}}_{max}$ relative humidity, sunshine hours, and average wind speed serve as subseries ${X}_{i}= \left\{ {X}_{i}(k)k=1,2,\ldots ,n \right\} ,$i = 1 , 2, …, *m*; *m* is the number of samples; k is the k-column sample; and n is the number of subseries.

Step 2: We determine the dimensionless treatment of relevant factors. In the system, the impact factors may differ in magnitude, making it difficult to compare them and draw the correct conclusions when comparing them. Therefore, data such as average temperature, wind speed, sunshine hours, and crop water requirements should be dimensionless to facilitate weighting and comparison.

(4) \begin{eqnarray*}{\mathrm{y}}_{\mathrm{k}}= \frac{{\mathrm{y}}_{\mathrm{k}}}{{\mathrm{y}}_{1}} {\mathrm{y}}_{\mathrm{k}}= \frac{{\mathrm{y}}_{\mathrm{k}}}{{\mathrm{y}}_{1}} \end{eqnarray*}

(5) \begin{eqnarray*}{X}_{ik}^{{}^{{^{\prime}}}}= \frac{{X}_{ik}^{{}^{{^{\prime}}}}}{{X}_{i1}^{{}^{{^{\prime}}}}} .\end{eqnarray*}

The above equation has 1 ≤ i ≤ n, and *k* is a sample of k columns.

Step 3: We determine the extremes of the poles.

(6) \begin{eqnarray*}{\mathop{\min \nolimits }\nolimits }_{i=1}^{n}{\mathop{\min \nolimits }\nolimits }_{k=1}^{m} \left\vert {y}_{k}^{{^{\prime}}}-{x}_{ik}^{{^{\prime}}} \right\vert \end{eqnarray*}

(7) \begin{eqnarray*}{\mathop{\max \nolimits }\nolimits }_{i=1}^{n}{\mathop{\max \nolimits }\nolimits }_{k=1}^{m} \left\vert {y}_{k}-{x}_{ik} \right\vert .\end{eqnarray*}

Step 4: We calculate the correlation coefficient of each subsequence with the corresponding element of each parent sequence.

(8) \begin{eqnarray*}{\zeta }_{ \left( k \right) }= \frac{\min _{i}\min _{k} \left\vert {y}_{k}^{{^{\prime}}}-{x}_{ik} \right\vert +p\cdot \max _{i}\max _{k} \left\vert {y}_{k}^{{^{\prime}}}-{x}_{ik} \right\vert }{ \left\vert {y}_{k}^{{^{\prime}}}-{x}_{ik}^{{^{\prime}}} \right\vert +p\cdot \max _{i}\max _{k} \left\vert {y}_{k}^{{^{\prime}}}-{x}_{ik}^{{^{\prime}}} \right\vert } .\end{eqnarray*}

The above equation takes p as 0.5, and 0 < p < 1 is the discrimination factor.

Step 5: Relevance is calculated.

(9) \begin{eqnarray*}r= \frac{1}{m} {\mathop{\sum \nolimits }\nolimits }_{k=1}^{m}\zeta (k).\end{eqnarray*}

### BiLSTM-CNN-Attention prediction model structure

**Figure 2:**
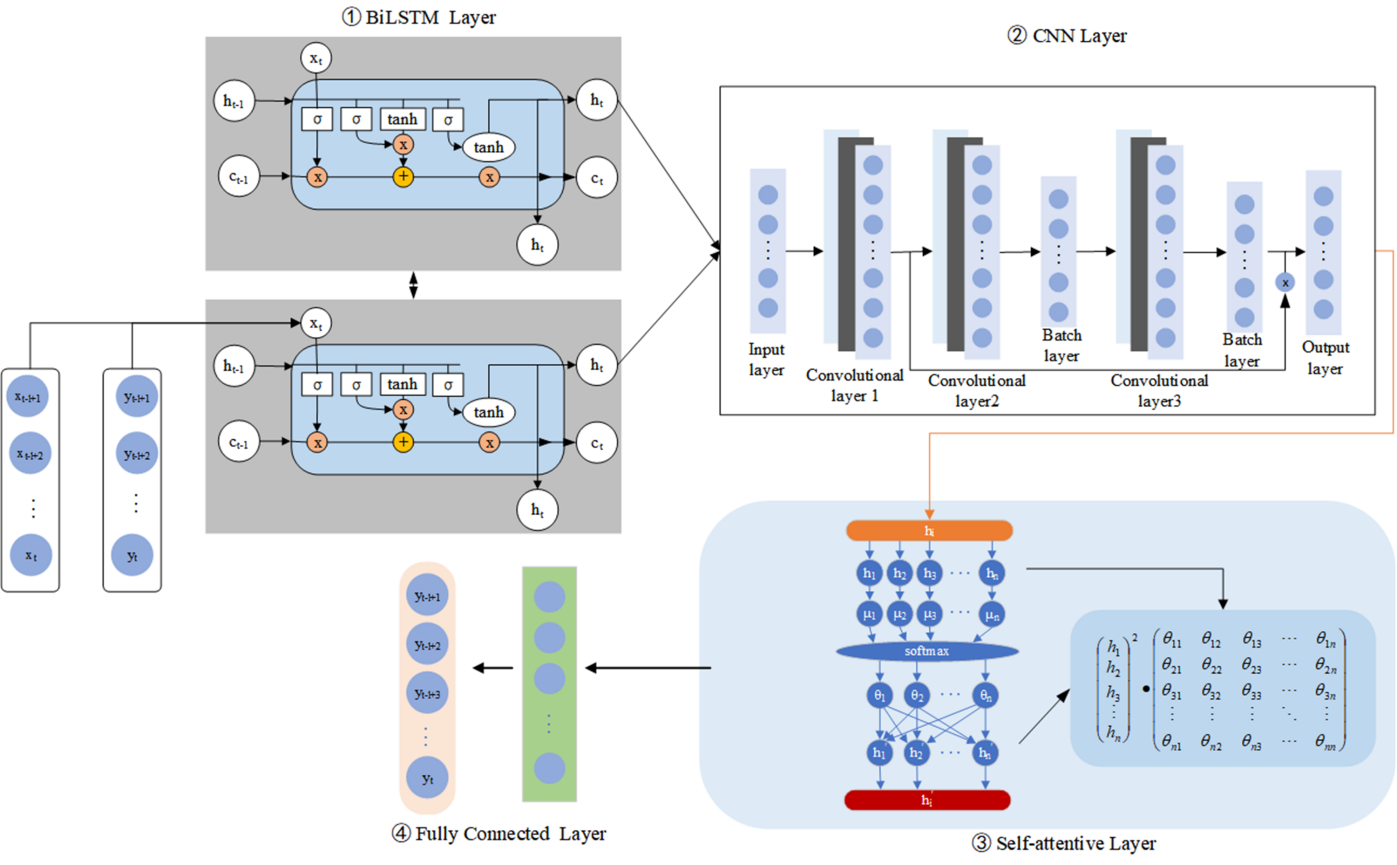
Flow chart for predicting irrigation water requirements for crops.

This study proposes a crop irrigation prediction method based on the BiLSTM-CNN-Attention model, and the specific implementation is shown in Fig. 2. We construct a BiLSTM-CNN-Attention model, and the data are first processed by a BiLSTM layer and a CNN layer to exploit the sequential coding capabilities of BiLSTM better. However, using the CNN layer first can lead to information loss about the data sequence. This model design can improve the accuracy of crop irrigation prediction.The technique involves pre-processing and splitting the collected irrigation data into training and test sets.

 This study also uses the BiLSTM model to extract the time series information by combining the model with three layers of 1D convolutions to construct a CNN framework for automatically extracting internal features from irrigation volume data. The convolutional layers efficiently remove the nonlinear local features of the irrigation volume data. Following the first two convolutional layers, batch normalization is also introduced to reduce the input bias by normalizing the batch samples’ net input. Then, we add residual join after the first and third convolutional layers to address the gradient vanishing and weight matrix degradation issues. Using the model design can improve the extraction of features from irrigation data and the accuracy of crop irrigation prediction.

The method utilized features extracted from the CNN layer to improve the attention mechanism. By applying the attention mechanism, the temporal information extracted from the CNN layer is weighted, helping to uncover deep temporal correlations and making more effective use of the time-series properties of the irrigation data. The attention mechanism also reduces the loss of historical information. It highlights information at key historical time-points, reducing redundant data’s impact on the irrigation volume prediction results. Next, the output of the attention layer is used as input to the fully connected layer, which produces the final irrigation volume prediction result. This approach takes full advantage of the time series information and reduces unnecessary interference factors, resulting in more accurate and predicting results.

The method also introduces a random deactivation (Dropout) technique to prevent the occurrence of over-fitting. Studies have used stochastic inactivation techniques to improve the generalization of models’ performance and reduce the training time. This technique improved model performance and efficiency during training. Regarding network parameter optimization, the method uses the adaptive moment estimation optimization algorithm (Adam) to update the network parameters of each layer. However, we used the MSE as the loss function. Finally, we saved the trained BiLSTM-CNN-Attention model and verified its effectiveness using a test set. We identified the model shortcomings by analyzing the irrigation prediction results and optimized the predictive models continuously. We followed this procedure to enhance its robustness and improve the predictive model’s performance.

Local sensing and weight sharing of CNN can significantly reduce the number of parameters and improve the model efficiency. These convolutional and pooling layers play crucial roles in this process. Each convolutional layer contains multiple kernels, and features are calculated using Eq. (10). The convolutional layer extracts the data features, but the feature dimension is steep. Adding a pooling layer after the convolutional layer can effectively reduce the feature dimension and contribute to the training cost.

(10) \begin{eqnarray*}{l}_{t}=\mathit{tanh} \left( {x}_{t}\ast {k}_{t}+{b}_{t} \right) \end{eqnarray*}

where *l*_*t*_ is the output value after convolution; tanh is the activation function ; *x*_*t*_ is the input vector; *k*_*t*_ is the weight of the convolution kernel; and *b*_*t*_ is the bias of the convolution kernel.

RNNs are either many-to-one or many-to-many with variable input and output lengths. RNNs are suitable for small-scale problems. Large-scale problems require a Transformer, and RNNs are not ideal for this application.

**Figure 3:**
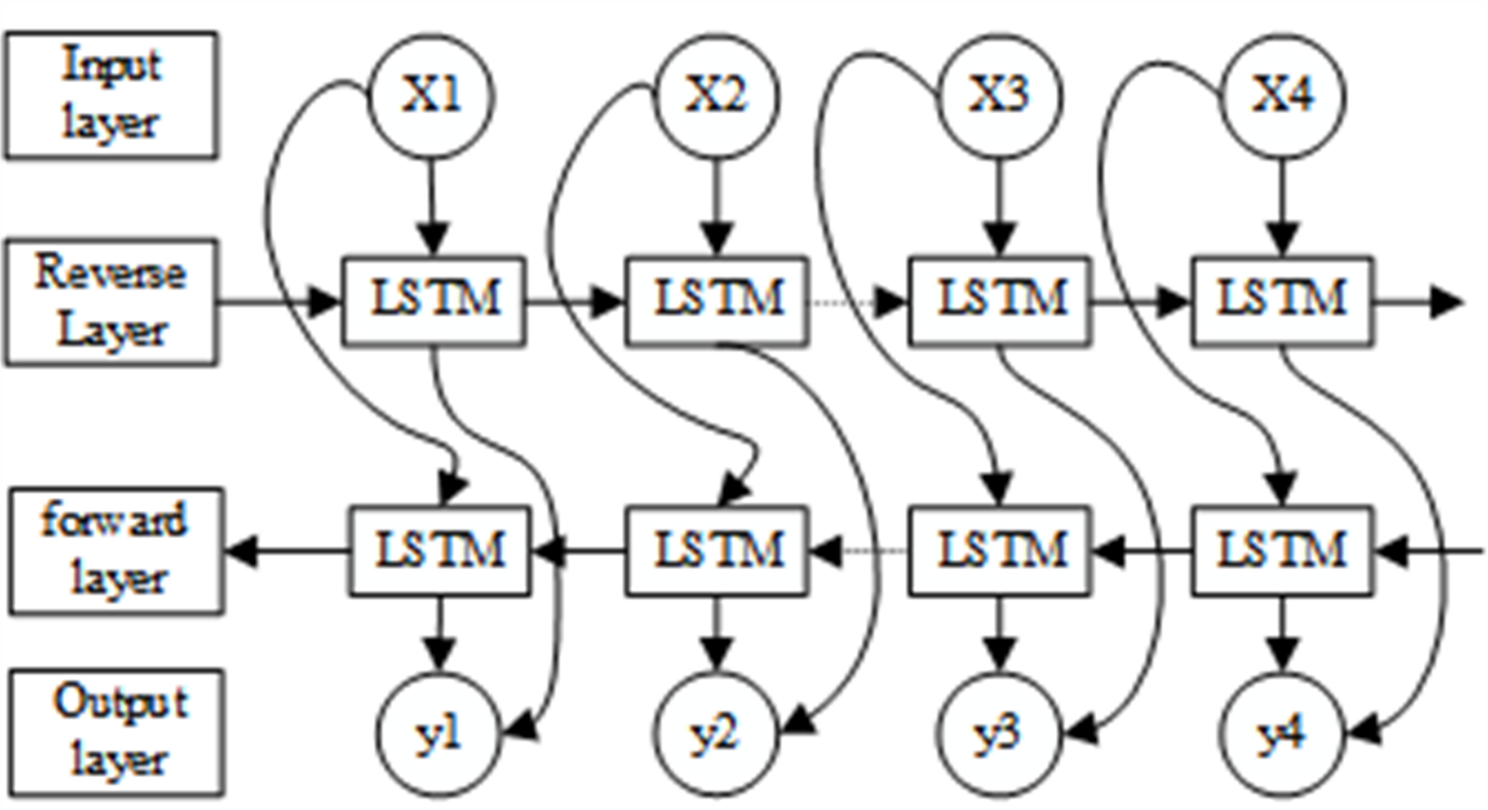
Structure of the BiLSTM-CNN-Attention model.

Moreover, LSTM only considers forward data dependencies. Therefore, BiLSTM uses LSTM networks in two opposite directions, front and back, to obtain complete information. The model also processes the input data and determines the current input by processing the data dependencies in both directions, as shown in Fig. 3. Our experiments demonstrate that the model performs better when backward data dependencies are considered. This approach improves the expressiveness of the model without requiring more data and reduces the risk of underfitting by re-using weights.

The LSTM calculation results are as follows:

In the first step, the LSTM determines the information that can be passed through the “cell state”. This decision is controlled by the “forget gate” layer (forgetting layer) through the sigmoid cell. The sigmoid function outputs a value between (0,1), filtering the information passed at the last moment and the information entered at the current moment. The calculation is shown in Eq. (11):

(11) \begin{eqnarray*}{f}_{t}=\sigma \left( {W}_{xf}{x}_{t}+{W}_{hf}{h}_{t-1}+{b}_{f} \right) .\end{eqnarray*}

The second step uses the “input gate” layer (input layer) to generate the data that can be updated when employing sigmoid cells. The calculation is shown in Eqs. (12), (13), (14) and (15):

(12) \begin{eqnarray*}{\mathrm{i}}_{\mathrm{t}}=\sigma \left( {\mathrm{W}}_{\mathrm{xi}}{\mathrm{x}}_{\mathrm{t}}+{\mathrm{W}}_{\mathrm{hi}}{\mathrm{h}}_{\mathrm{t}-1}+{\mathrm{b}}_{\mathrm{i}} \right) \end{eqnarray*}

(13) \begin{eqnarray*}{\mathrm{c}}_{\mathrm{t}}={\mathrm{f}}_{\mathrm{t}}{\mathrm{c}}_{\mathrm{t}-1}+{\mathrm{i}}_{\mathrm{t}}\text{tanh} \left( {\mathrm{W}}_{\mathrm{xc}}{\mathrm{x}}_{\mathrm{t}}+{\mathrm{W}}_{\mathrm{hc}}{\mathrm{h}}_{\mathrm{t}-1}+{\mathrm{b}}_{\mathrm{c}} \right) \end{eqnarray*}

(14) \begin{eqnarray*}{\mathrm{o}}_{\mathrm{t}}=\sigma \left( {\mathrm{W}}_{\mathrm{xo}}{\mathrm{x}}_{\mathrm{t}}+{\mathrm{W}}_{\mathrm{ho}}{\mathrm{h}}_{\mathrm{t}-1}+{\mathrm{b}}_{\mathrm{c}} \right) \end{eqnarray*}

(15) \begin{eqnarray*}{\mathrm{h}}_{\mathrm{t}}={\mathrm{o}}_{\mathrm{t}}\text{tanh} \left( {\mathrm{c}}_{\mathrm{t}} \right) \end{eqnarray*}

where *x*_*t*_ is the input; *i*_*t*_ is the input gate; *f*_*t*_ is the forgetting gate; *c*_*t*_ is the state of the cell at the moment t; *o*_*t*_ is the cell state at the output at moment t; *h*_*t*_ is the production at moment t; *σ* is the sigmoid function; tanh is the hyperbolic tangent function; *b*_*i*_, *b*_*f*_, *b*_*c*_, *b*_*o*_ are the paranoid vectors; and *W*_*xi*_, *W*_*hi*_, *W*_*xf*_, *W*_*hf*_, *W*_*xc*_, *W*_*hc*_, *W*_*xo*_, *W*_*ho*_ are the weighting factors.

When inputting computational irrigation data into a neural network, it is common to encounter vectors of varying sizes, each with specific relationships between them. These differences in vectors can pose a challenge. Therefore, it is challenging to sufficiently exploit these relations during practical training, resulting in poor model results. Introducing the attention mechanism allows critical information to be selected and highlights essential inputs. This study used a forward-attentive model, learning through attention weights, which involved adding a feed-forward network to the existing network structure. The attention weights of the feed-forward network are functions of the hidden state values of the encoder and decoder. Therefore, we used a feed-forward network trained jointly with the previous network architecture.

(16) \begin{eqnarray*}{\mathrm{e}}_{\mathrm{t}}=\text{tanh} \left( {\mathrm{h}}_{\mathrm{t}} \right) \end{eqnarray*}

(17) \begin{eqnarray*}{\alpha }_{\mathrm{t}}= \frac{\exp \nolimits \left( {\mathrm{e}}_{\mathrm{t}} \right) }{{\mathop{\sum \nolimits }\nolimits }_{\mathrm{t}=1}^{\mathrm{m}\sum \left( {\mathrm{e}}_{\mathrm{t}} \right) }\exp \nolimits } .\end{eqnarray*}

The generated attention weights are assigned to the corresponding hidden layer *h*_*t*_ so that the attention weights generated by the model are functional. Its *h*_*t*_ weighted average and the consequences *α*_*t*_ are shown in Eq. (18).

(18) \begin{eqnarray*}{\mathrm{c}}_{\mathrm{t}}=\sum _{\mathrm{t}=1}^{\mathrm{m}}{\alpha }_{\mathrm{ t}}\cdot {\mathrm{h}}_{\mathrm{t}}.\end{eqnarray*}

### BiLSTM-CNN-Attention training process

**Figure 4:**
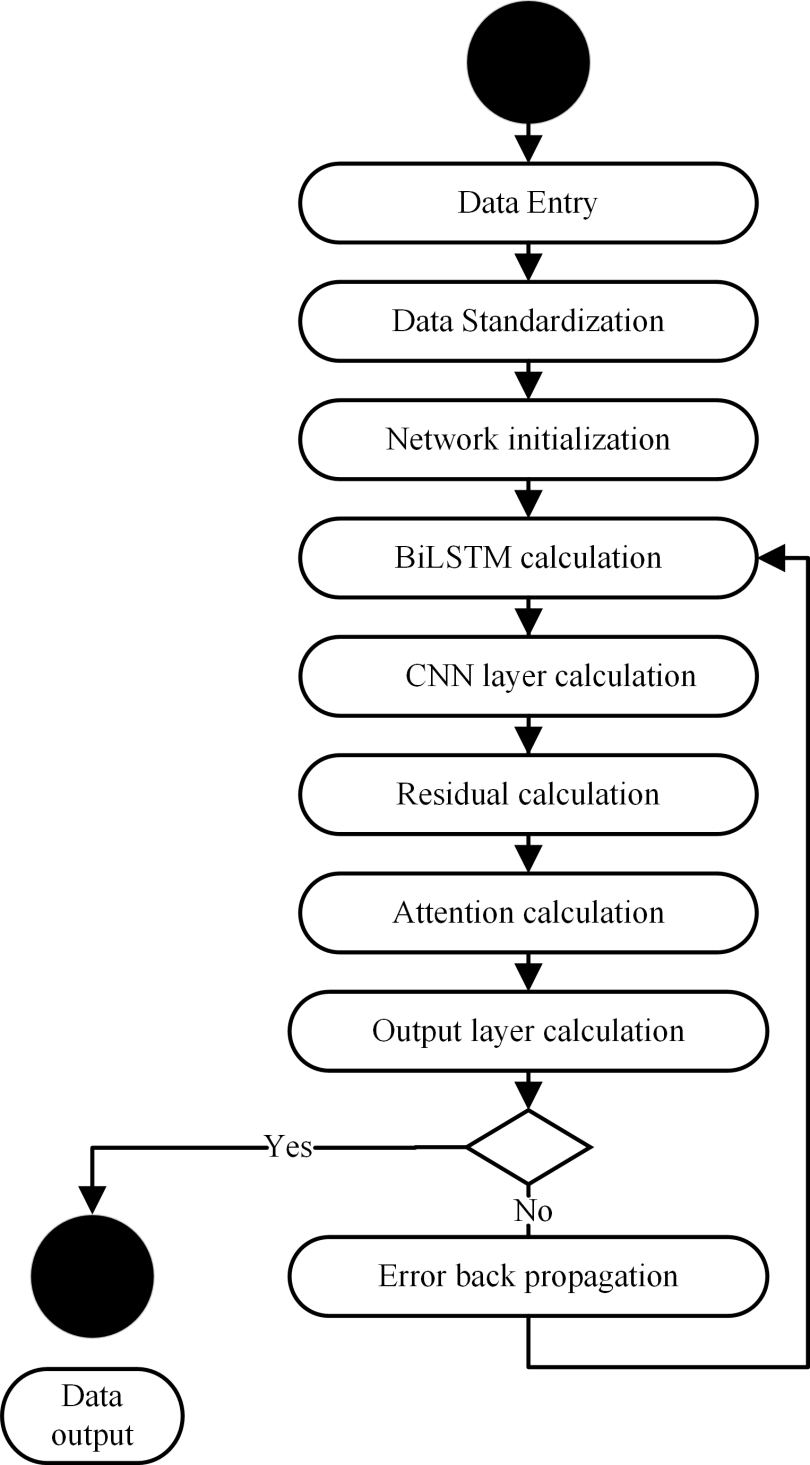
BiLSTM memory cell structure.

The BiLSTM-CNN-Attention training process is shown in Fig. 4.

The main steps of the BiLSTM-CNN-Attention training are as follows.

(1) Input data: The first step is to input data for BiLSTM-CNN-Attention training.

(2) Input data normalization: We used the z-score normalization method to normalize the input data to train the model due to the significant variation in the input data.

(3) Network initialization: We initialize the BiLSTM-CNN-Attention layer weights and biases.

(4) BiLSTM layer calculation: Then, we extract the time series information from the irrigation data, calculate the input data of the CNN layer through the hidden layer of the BiLSTM layer, and obtain the output value.

(5) CNN layer calculation: The input data are sequentially passed through 3 CNN layers within a convolutional layer to perform feature extraction on the input data and obtain the output value.

(6) Residual calculation: The residuals are accumulated from the output values of the first convolutional layer after passing through the BiLSTM layers and the third convolutional layer normalized by batch normalization.

(7) Attention layer calculation: The attention layer calculates the output data of the CNN layer to obtain the output value.

(8) Output layer calculation: We calculate the output value of the attention layer to obtain the model output value.

(9) Calculation error: We compared the output value calculated with the actual value of this data set. This process enables us to calculate the corresponding error and assess our model’s accuracy.

(10) We determine if the end conditions for the prediction process are met: The requirements for a successful end are the completion of a predetermined number of cycles, a weight below a certain threshold, and a prediction error rate below a certain point. The training is completed if at least one of the end conditions is met. Otherwise, the movement continues.

(11) Error backpropagation: The calculated error is propagated in the opposite direction, updating the weights and biases of each layer and returning to step (4) to continue the network training.

### BiLSTM-CNN-attention training process

**Figure 5:**
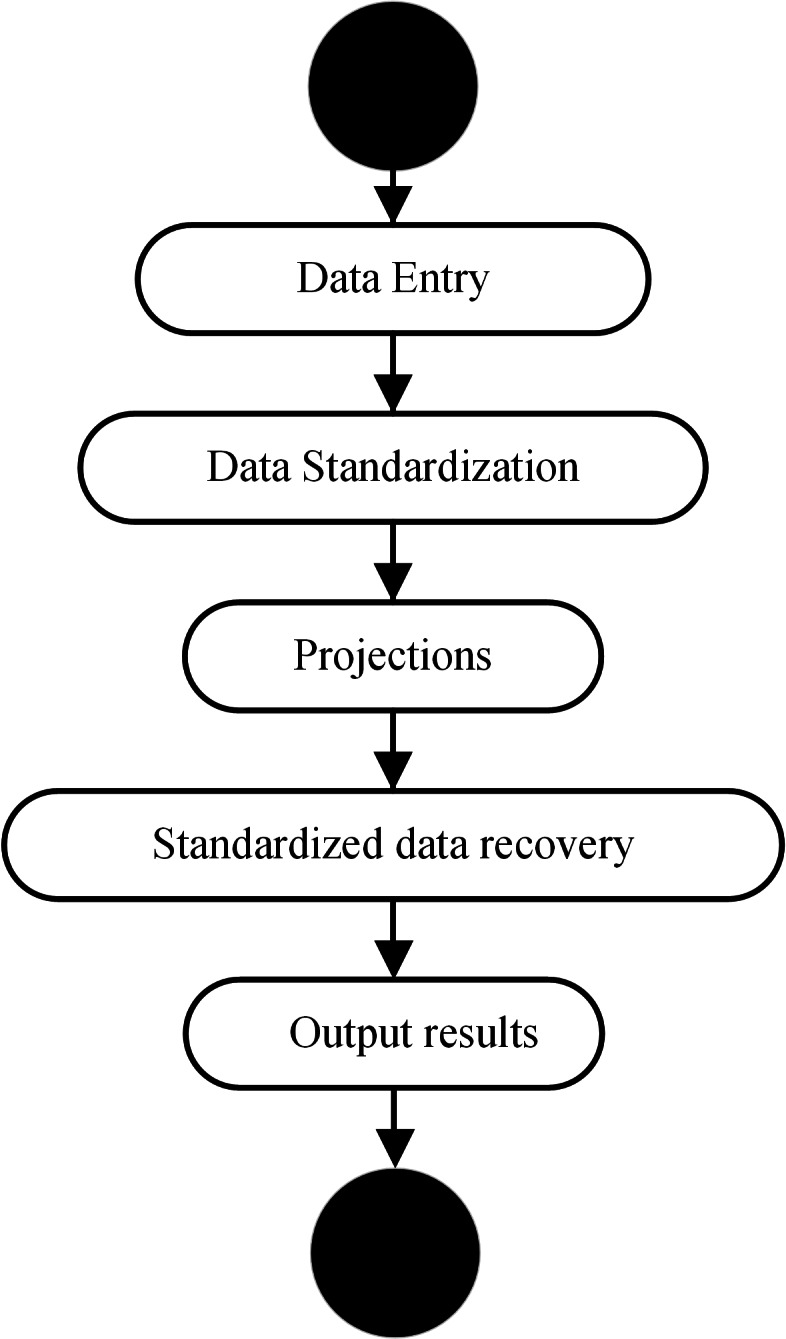
BiLSTM-CNN-Attention training flow chart.

BiLSTM-CNN-Attention is only possible if BiLSTM-CNN-Attention has been trained (Fig. 5).

The steps are as follows:

(1) Input data: We input data required for the forecast.

(2) Input data normalization: The input data are normalized according to (11).

(3) Prediction: The normalized data are fed into the trained BiLSTM-CNN-Attention to obtain the corresponding output values.

(4) Data normalization recovery: The output values obtained by BiLSTM-CNN-Attention are normalized, and the normalization formula is shown in Eq. (19):

(19) \begin{eqnarray*}{x}_{i}={y}_{i}\ast s+\bar {x}\end{eqnarray*}

where *x*_*i*_ is the normalized recovery value ; *y*_*i*_ is the output value of BiLSTM-CNN-Attention ; *s* is the input data’s standard deviation; and $\overline{X}$ is the mean of the input data *x*.

(5) Output results: We output the recovered results to complete the prediction process.

### Evaluation indicators

We evaluated the prediction accuracy of the BiLSTM-CNN-Attention model by comparing it with other models, such as RNN, LSTM, BiLSTM, and BiLSTM-CNN. This process helps to predict the amount of irrigation for spring corn from May 1, 2020 to September 30, 2022 in Changchun, Jilin Province, China. Then, we evaluated the proposed model performance using MAE, MSE, RMSE, and R^2^. The model performs better when MAE, MSE, and RMSE are smaller. However, a larger value of the R^2^ indicates a better fit of the model to the data.

The calculation formula is as follows:

(20) \begin{eqnarray*}MAE= \frac{1}{n} {\mathop{\sum \nolimits }\nolimits }_{i=1}^{n} \left\vert {\hat {y}}_{i}-{y}_{i} \right\vert \end{eqnarray*}

(21) \begin{eqnarray*}\mathrm{MSE}= \frac{1}{n} {\mathop{\sum \nolimits }\nolimits }_{i=1}^{n}{ \left( {\hat {y}}_{i}-{y}_{i} \right) }^{2}\end{eqnarray*}

where ${\hat {y}}_{i}$ is the predicted value and *y*_*i*_ is the actual value.

The calculation formula of *RMSE* is as follows:

(22) \begin{eqnarray*}RMSE=\sqrt{ \frac{1}{n} {\mathop{\sum \nolimits }\nolimits }_{i=1}^{n}{ \left( {\hat {y}}_{i}-{y}_{i} \right) }^{2}}\end{eqnarray*}

where ${\hat {y}}_{i}$ is the predicted value and *y*_*i*_ is the actual value.

The calculation formula of *R*^2^ isas follows:

(23) \begin{eqnarray*}{R}^{2}=1- \frac{ \left( {\mathop{\sum \nolimits }\nolimits }_{i=1}^{n}{ \left( {y}_{i}-{\hat {y}}_{i} \right) }^{2} \right) /n}{ \left( {\mathop{\sum \nolimits }\nolimits }_{i=1}^{n}{ \left( \overline{{y}_{i}}-{\hat {y}}_{i} \right) }^{2} \right) /n} \end{eqnarray*}

where ${\hat {y}}_{i}$ is the predicted value, *y*_*i*_ is the actual value, and ${\bar {y}}_{i}$ is the average value.

## Results and Analysis

Our experiments are performed on Windows 10, a 64-bit operating system, with an NVIDIA GeForce GTX 1060 6GB GPU, ATTENTION Ryzen 2700X processor, and 16GB RAM. Our goal is to demonstrate the accuracy of the BiLSTM-CNN-Attention prediction for irrigation. The experiments are also performed on the TensorFlow framework. Moreover, Python 3.6 was used for programming, using required libraries such as Numpy, Pandas, and OpenCV.

**Table 2:** Selected experimental data.

Date (UTC)	Highest Temperature 2 m(°C)	Minimum Temperature 2 m(°C)	Precipitation (mm)	Relative humidity (%)	Wind speed (m/s)	Hours of sunshine (h)	Water requirements (mm)
20000501	17.59	7.63	2.79	72.19	2.34	5.73	2.8
20000502	22.07	10.07	0.01	48.44	2.4	6.56	2.3
20000503	23.85	7.75	0.02	47.92	3.87	6.37	2.2
...	...	...	...	...	...	...	...
20200930	16.93	4.34	0	66.22	2.3	3.92	1.6149

**Table 3:** Selected text data.

Date (UTC)	Highest Temperature 2m(°C)	Minimum Temperature 2m(°C)	Precipitation (mm)	Relative humidity (%)	Wind speed (m/s)	Hours of sunshine (h)	Water requirements (mm)
20210501	15.26	2.72	0.00	57.42	2.76	6.29	1.3
20210502	18.29	8.23	0.48	47.78	2.89	6.07	1.2
20210503	20.64	11.25	1.06	37.48	6.38	4.73	1.5
...	...	...	...	...	...	...	...
20220930	26.51	9.1	25.04	75.83	5.2	4.15	2.7

### Data sources

The model requires daily meteorological data values and annual mean atmospheric CO2 concentrations. We obtained meteorological data from the WheatA Small Malt-Agricultural Meteorological Big Data System V1.5.4a. For the training set, we opted for irrigation data from May 1, 2000 to September 30, 2020. However, irrigation data has been designated for the independent prediction dataset from May 1, 2021 to September 30, 2022. Detailed information regarding the training data is presented in Table 2, while specifics concerning the prediction data are presented in Table 3.

**Figure 6:**
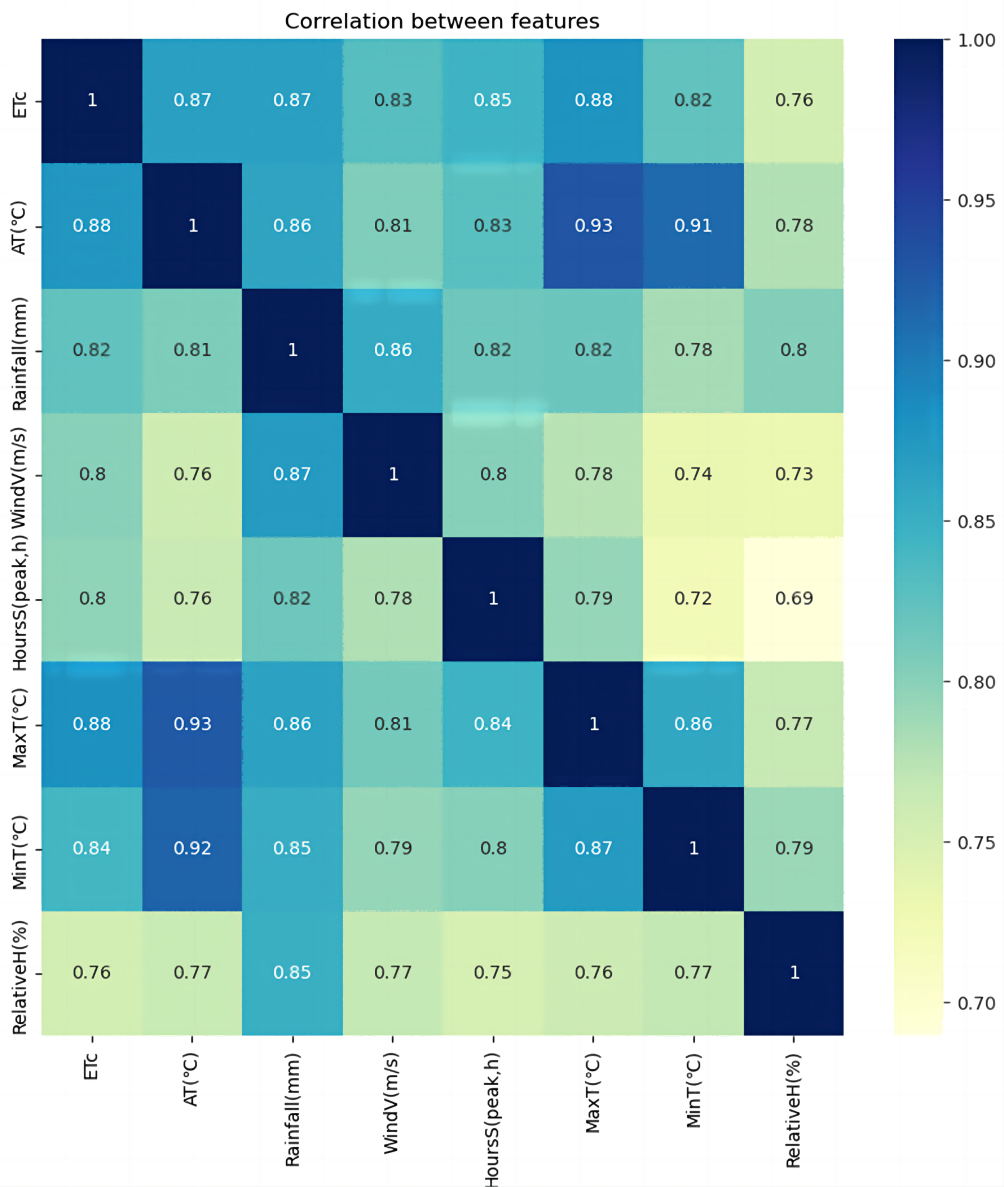
Correlation analysis.

The GCA has been utilized to analyze the correlation between each feature value from the daily monitored meteorological data in Changchun, Jilin Province, China. The data span from May 2000 to September 2020, providing a comprehensive understanding of the meteorological patterns in the region. The Pycharm environment has been employed to implement the GCA, as shown in Fig. 6.

**Table 4:** BiLSTM-CNN-attention prediction model parameter settings.

	Parameters	Parameter values
Input layer	Input Nodes Convolutional layers filters Convolutional layer kernel_size Convolutional layer activation function	11 64 3 Relu
CNN layer	Convolution layer padding Pooling layer pool_size Pooling layer padding Number of LSTM hidden cells	Same 1 same 8
BiLSTM layer	LSTM activation function	tanh
Attention layer Output layer	Activation functions Number of output nodes Time step Optimizers Learning Rate	Softmax 1 20 Adam 0.001
Training parameters	Loss function batch_size epochs	mean_squared_error 512 500

In Fig. 6, We used AT–average temperature, rainfall (mm)–rainfall, WindV (m/s)–average wind speed, HoursS (peak, hours)–hours of sunshine, MaxT (°C)–maximum temperature, MinT (°C)–minimum temperature, and RelativeH (%)–relative humidity for the correlation analysis. As shown in Table 4, the correlation between maximum temperature and crop water requirement is the best, with a correlation coefficient of 0.88. Average temperatures and rainfall subsequently showed the highest correlation with crop water requirements, with a correlation coefficient of 0.87. Wind speed and sunshine hours were also strongly correlated with crop water requirements, with coefficients ranging from 0.7 to 0.85. Figure 6 shows that the more significant the correlation between the influence factor and the target series, the darker the corresponding module color, indicating a closer relationship.

### Model parameter settings

Table 4 shows the parameters of the BiLSTM-CNN-Attention model set for this experiment, exhibiting that the training parameters are the same for all our methods, with epochs of 500, loss function MSE, optimizer Adam, time step of 20, and learning rate of 0.001.

### Results of the irrigation prediction model

The processed training set data were used to train CNN, RNN, LSTM, BiLSTM, CNN-LSTM, BiLSTM-CNN, and BiLSTM-CNN-Attention. The trained model was used to predict the test set data, and the valid values were compared with the predicted values.

**Figure 7:**
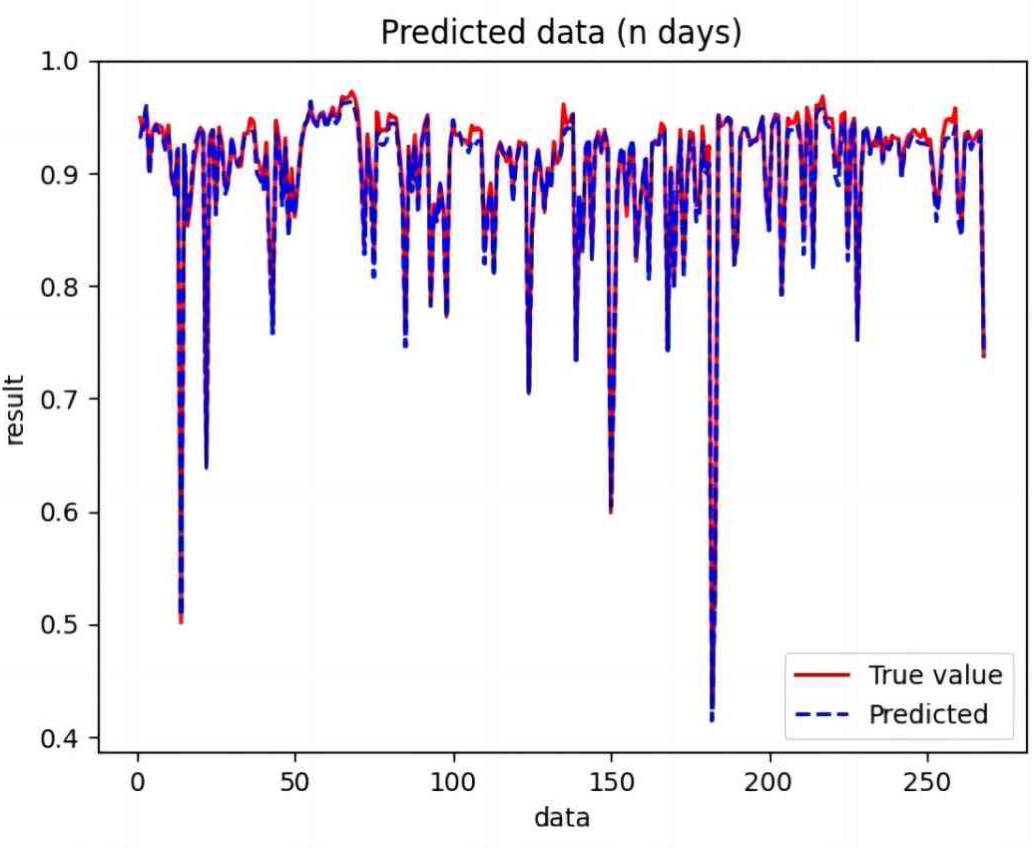
Comparison of CNN predicted and actual values.

**Figure 8:**
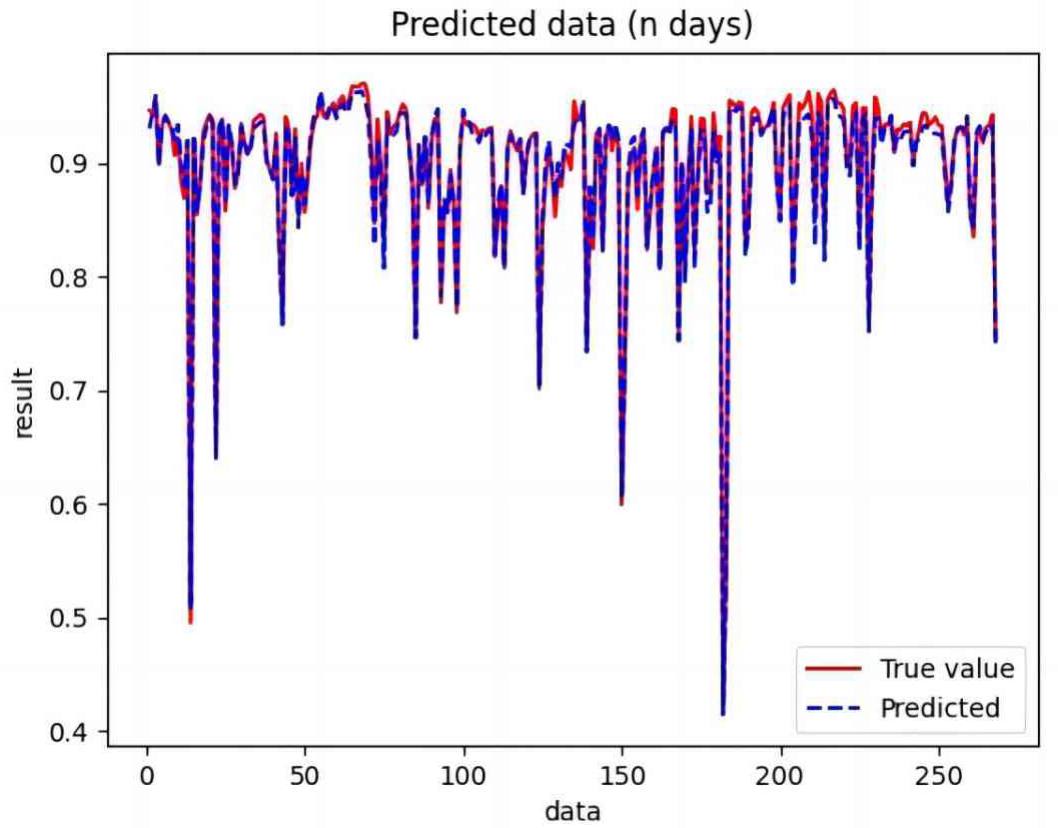
Comparison of RNN predicted and actual values.

**Figure 9:**
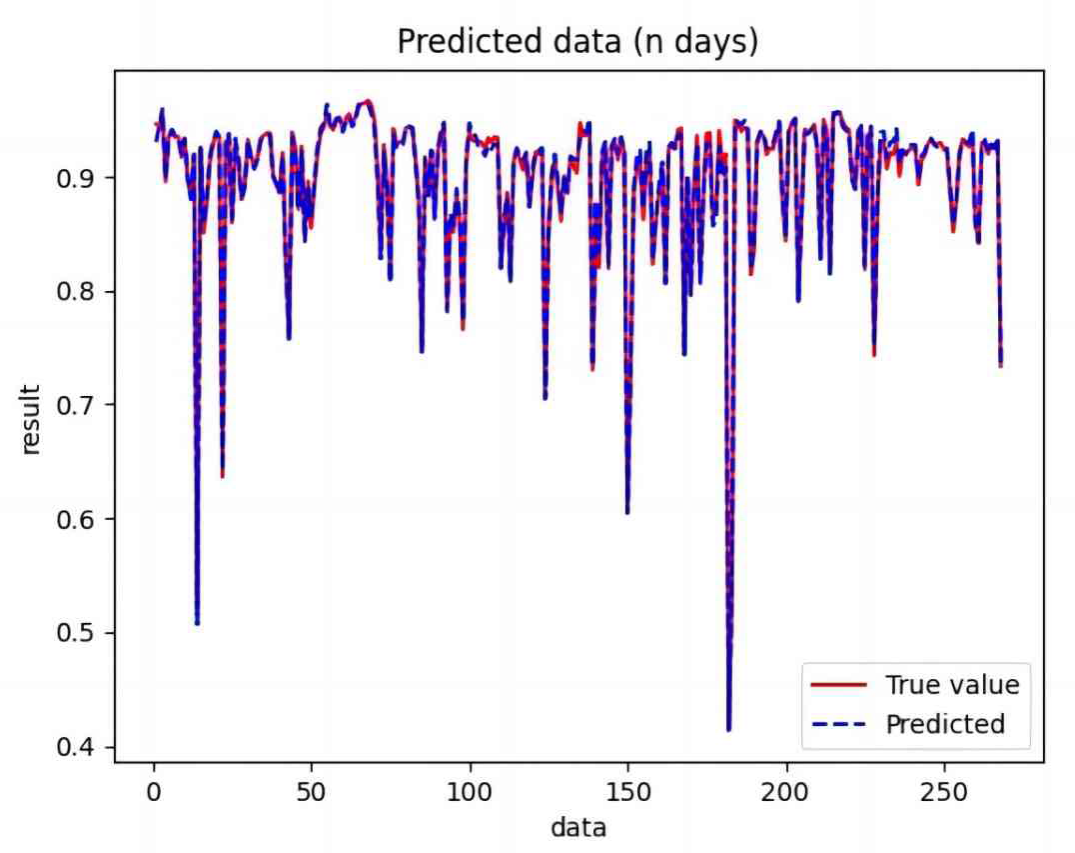
Comparison of LSTM predicted and actual values.

**Figure 10:**
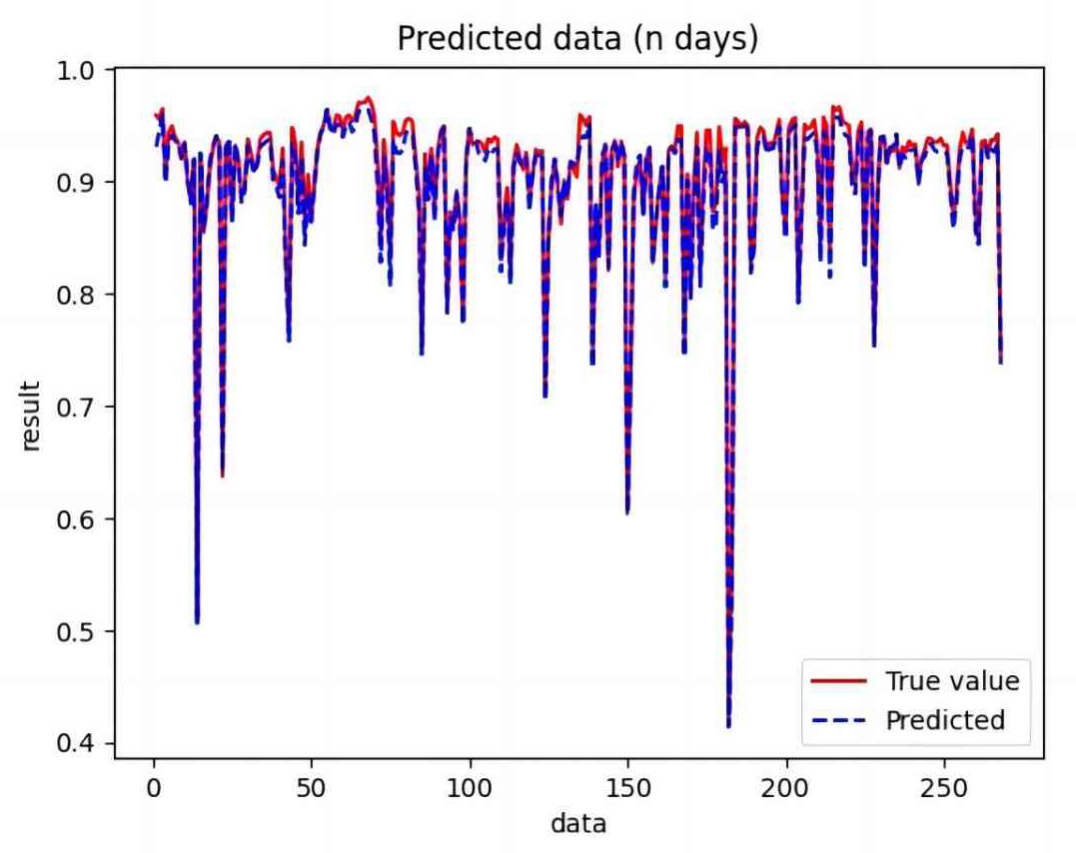
Comparison of BiLSTM predicted and actual values.

**Figure 11:**
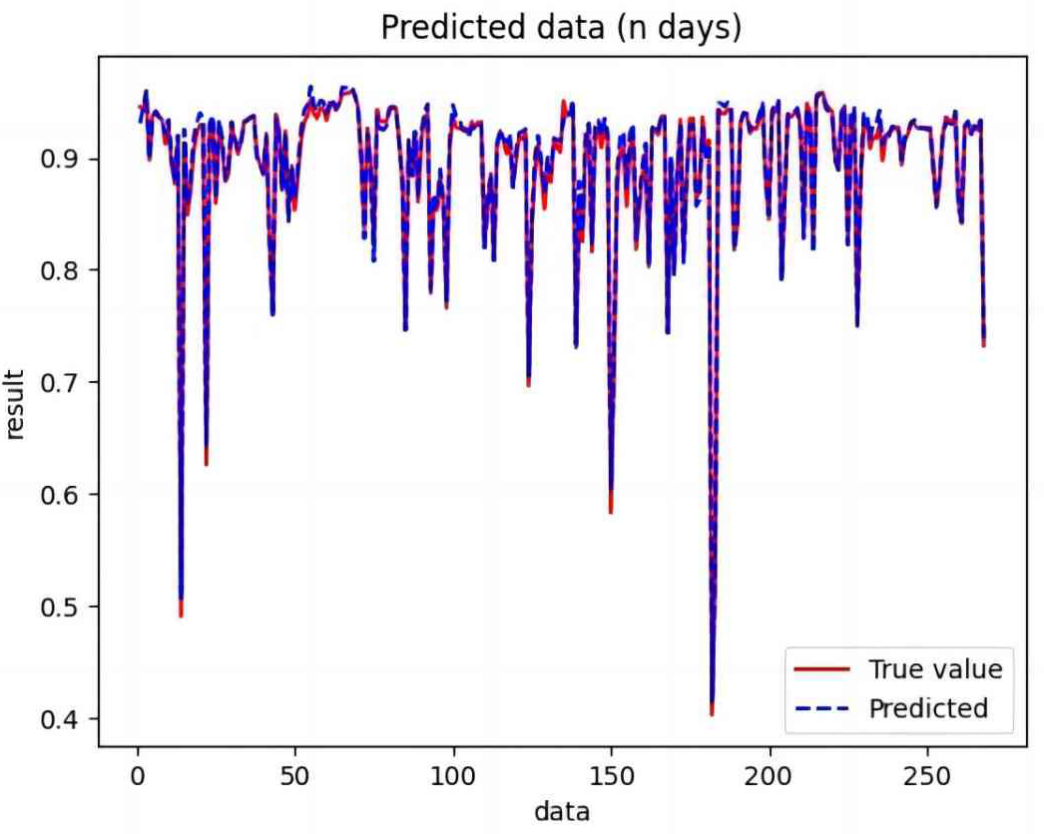
Comparison of BiLSTM-CNN predicted and actual values.

**Figure 12:**
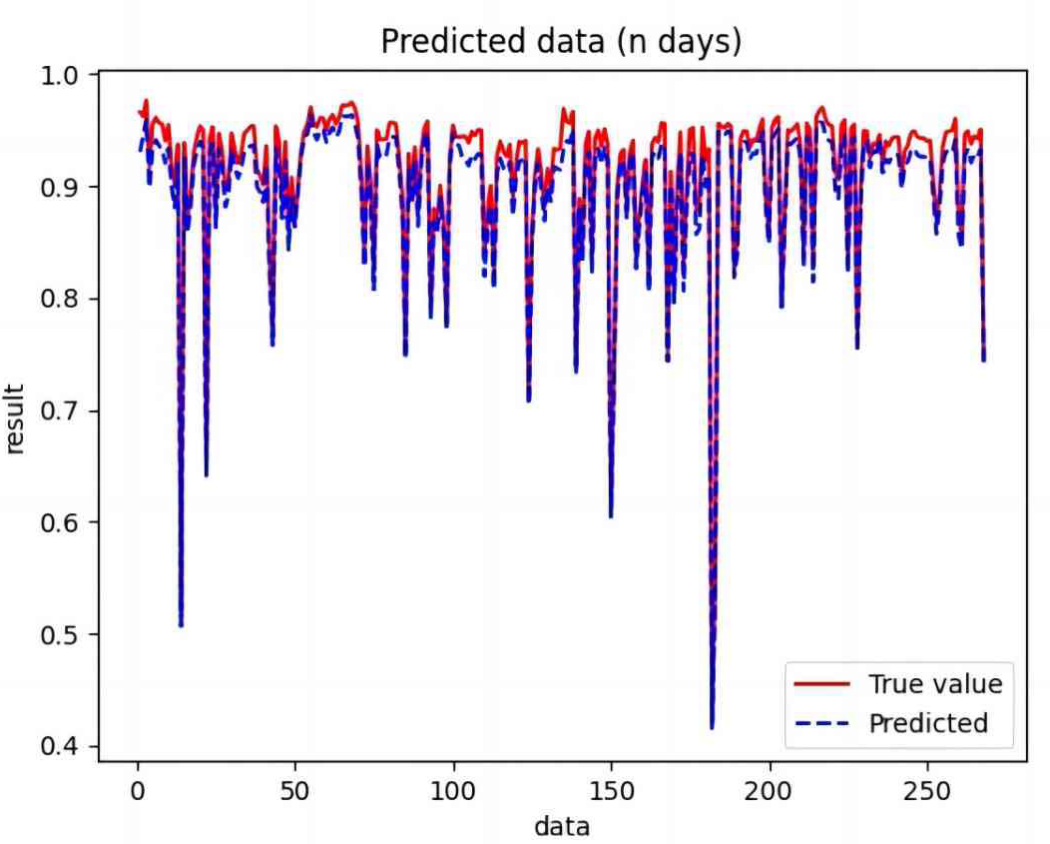
Comparison of BiLSTM-CNN-Attention predicted and actual values.

Based on the analysis of Figs. 7–12, the fold fit between the predicted and fundamental values of six prediction methods follows from highest to lowest: BiLSTM-CNN-Attention, BiLSTM-CNN, BiLSTM, LSTM, CNN, and RNN. Notably, RNN had the lowest fold fit. Based on each method’s predicted and actual values, the evaluation error indicators are calculated by comparing the results of the six methods shown in Table 5.

**Figure 13:**
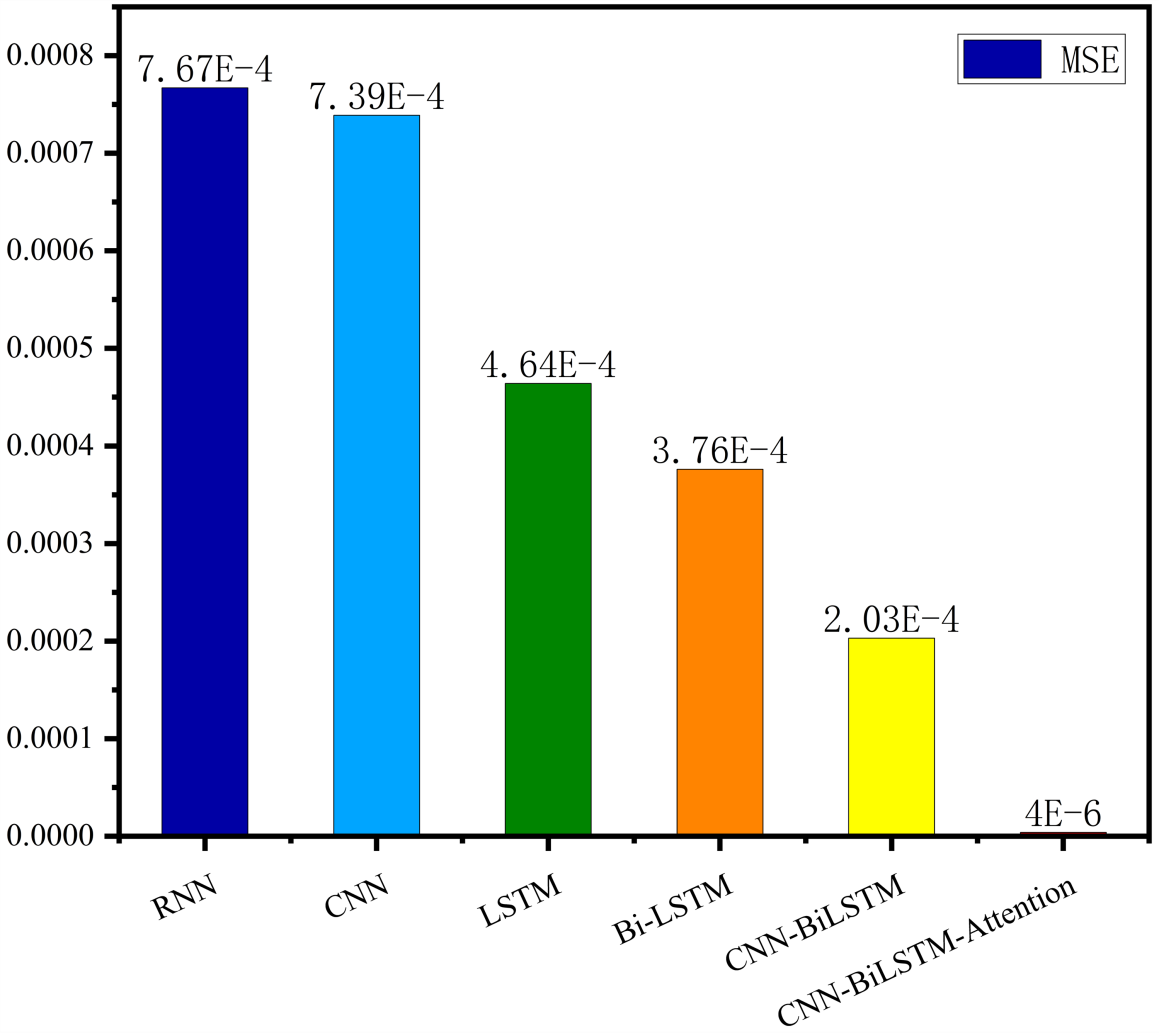
Correlation analysis.

**Figure 14:**
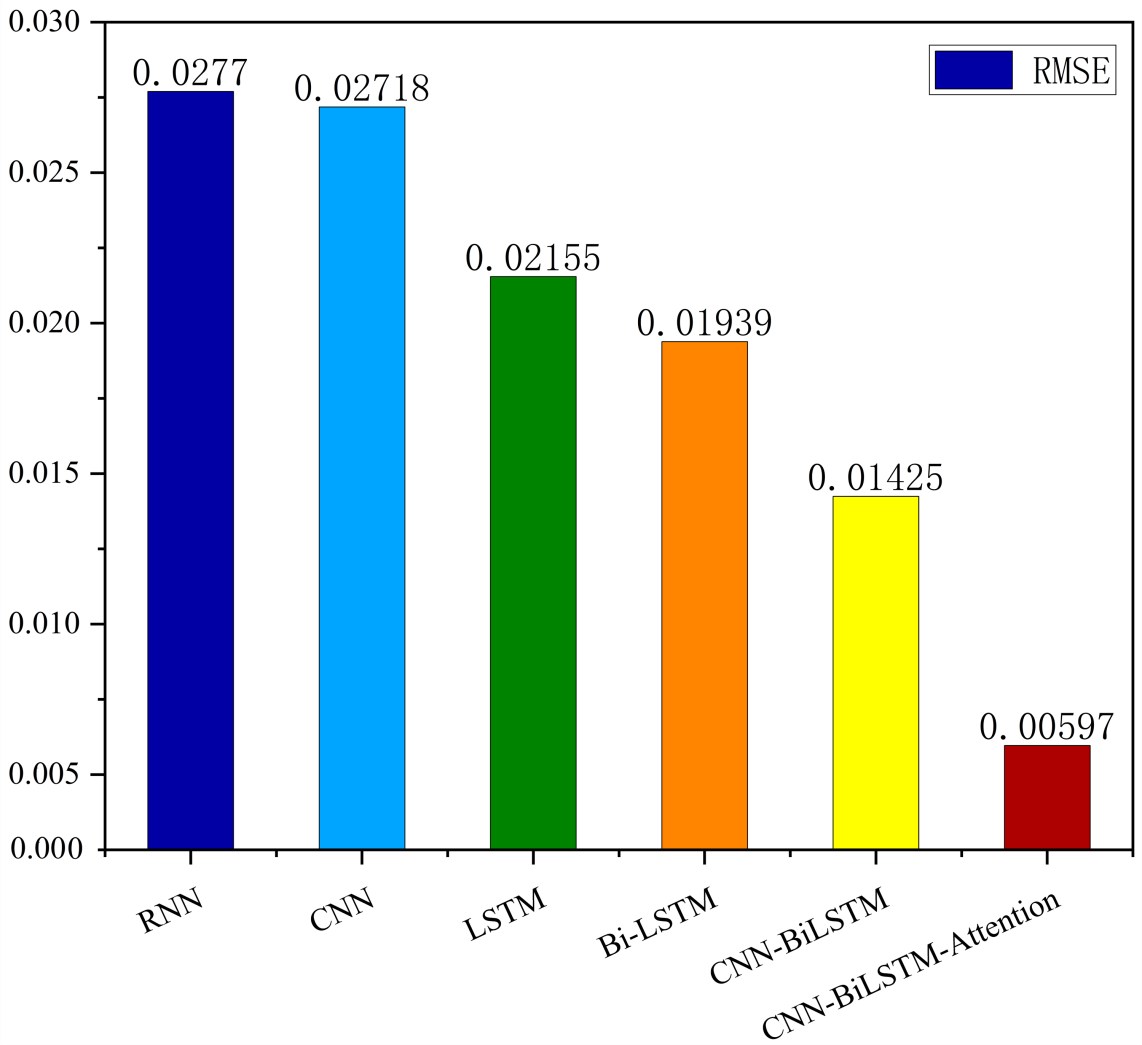
Comparison of RNN, CNN, LSTM, BiLSTM, BiLSTM-CNN, BiLSTM-CNN-Attention, predicted and actual values.

**Figure 15:**
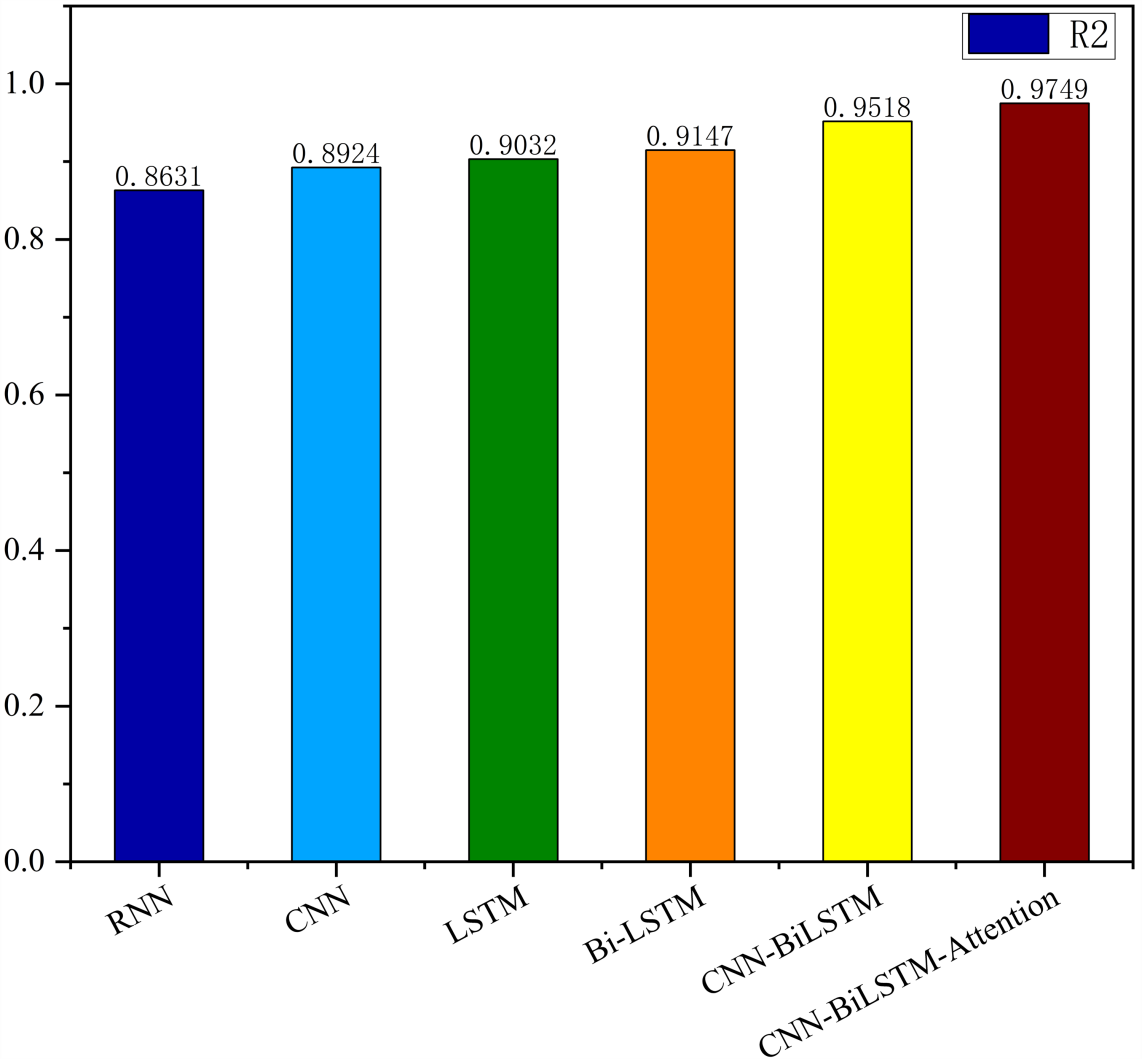
Comparison of MSE results for different models.

**Figure 16:**
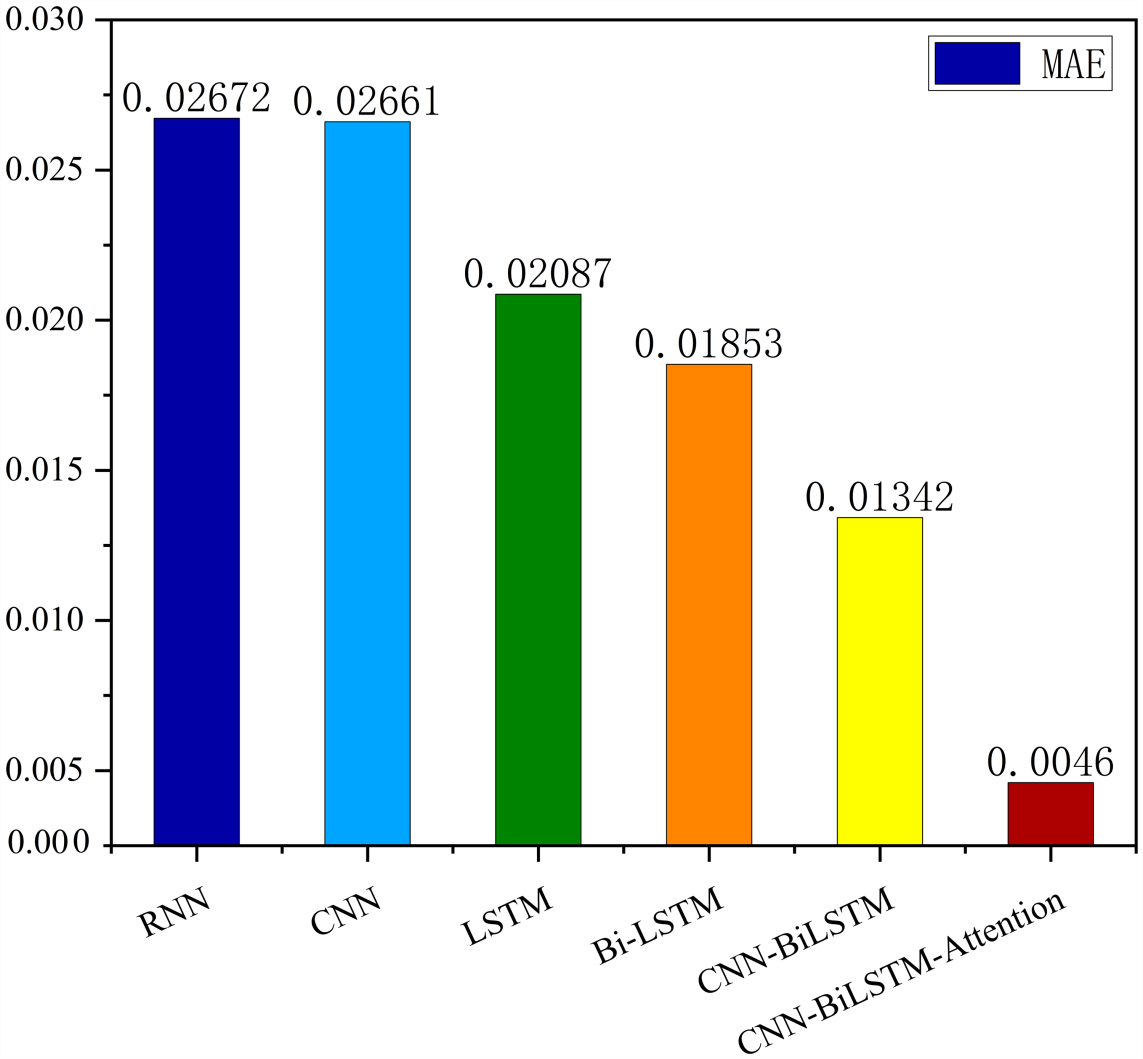
Comparison of RMSE results for different models.

The MSE, RMSE, and MAE of RNN are the largest, and R^2^ is the smallest, as shown in Table 6 and Figs. 13–16. Moreover, MSE, RMSE, and MAE of BiLSTM-CNN-Attention are the smallest, and R^2^ is the largest and closest to 1. The prediction performance of the six methods is in descending order: BiLSTM-CNN-Attention, BiLSTM-CNN, BiLSTM, LSTM, CNN, and RNN. -CNN, BiLSTM, LSTM, CNN, RNN. However, LSTM has smaller MSE, RMSE, and MAE and larger R^2^ than RNN. Its MAE (0.020867 *vs.* 0.026724) was 21.59% lower, and its MSE (0.000464 *vs.* 0.000767) was 39.5% lower than RNN. Its RMSE (0.021549 *vs.* 0.027701) was 22.2% lower than RNN. Its R^2^ is 4.01% larger than RNN. Therefore, LSTM outperforms RNN. BiLSTM reduces MAE from 0.020867 to 0.018530, MSE from 0.000464 to 0.000376, RMSE from 0.021549 to 0.019387, R^2^ from 0.9032 to 0.9147 compared with LSTM, indicating that the prediction accuracy of BiLSTM has been improved compared with LSTM.

The BiLSTM decreases in MAE and RMSE and increases in R^2^ compared to the BiLSTM after CNN layers. Moreover, MAE decreased from 0.018530 to 0.013423. MSE decreased from 0.000376 to 0.000203, and RMSE decreased from 0.019387 to 0.014247. R^2^ increased to 0.9518. The results show that BiLSTM-CNN-Attention performs best among the six methods. Its MAE is 0.004599, RMSE is 0.005968, and R^2^ is 0.9926. Therefore, among the six methods, the proposed BiLSTM-CNN-Attention method exhibits a higher accuracy in predicting the future crop irrigation amount, providing a reference for agricultural workers to make correct irrigation decisions.

We are currently performing additional verification to validate the optimized LSTM model. In addition to investigating irrigation practices for spring corn in agricultural bases in Changchun City, Jilin Province, we are currently studying the irrigation conditions for greenhouse tomatoes at the “Good Yunlai” agricultural base in Yuncheng County, Heze City, Shandong Province. This extended research aims to verify the model’s applicability under different geographical and crop conditions.

## Conclusions

This study presents an accurate irrigation volume prediction method based on an optimized LSTM model and demonstrates its importance and effectiveness in irrigation decision-making and crop production management. Therefore, we analyze meteorological data and model the correlation between meteorological data and crop water requirements. We also conducted an extrapolation test using the AquaCrop model to determine the optimal irrigation strategy. For temporal features of crop irrigation volume data, we propose a BiLSTM-CNN-Attention method using six meteorological indicators such as precipitation, mean temperature, maximum temperature, minimum temperature, sunshine hours, and mean wind speed as inputs. The proposed method fully integrates the BiLSTM model, CNN model, and attention mechanism to exploit spatiotemporal features and sequence dependencies, improving prediction accuracy and reliability. Experimental results show that the BiLSTM-CNN-Attention method presents significant advantages over alternative methods for predicting irrigation volume. The procedure performs best in terms of MSE, RMSE, and MAE metrics, with an R^2^ value of 0.9749, close to one after comparing the predictions with appropriate values. Compared with the RNN, CNN, LSTM, and BiLSTM models, the proposed method improves the R^2^ values by 11.468%, 8.462%, 7.355%, and 6.175%, respectively. Compared to the BiLSTM-CNN model, the BiLSTM-CNN-Attention method improves the R^2^ value by 2.369%.

**Table 5:** Calculation of correlations.

Parameters	Average temperature (°C)	Rainfall (mm)	Wind speed (m/s)	Relative humidity (%)	Maximum temperature (°C)	Lowest temperature (°C)	Hours of sunshine (h)
Relevance	0.87	0.87	0.83	0.85	0.88	0.82	0.76

**Table 6:** Comparison of evaluation error indicators.

Models	MSE	RMSE	R^2^	MAE
RNN	0.000767	0.027701	0.8631	0.026724
CNN	0.000739	0.027180	0.8924	0.026611
LSTM	0.000464	0.021549	0.9032	0.020867
Bi-LSTM	0.000376	0.019387	0.9147	0.018530
BiLSTM-CNN	0.000203	0.014247	0.9518	0.013423
BiLSTM-CNN-Attention	0.000004	0.005968	0.9749	0.004599

Our results show that achieving robust prediction accuracy using a single network is difficult. Still, by complicating the network structure, introducing BiLSTM, CNN, and attention mechanisms, prediction accuracy can be significantly improved. The BiLSTM-CNN-Attention approach has promising applications in predicting crop irrigation and can be a valuable reference for agricultural workers. We use this approach as a reference standard for maximizing the return on investment. Therefore, this study provides practical experience in studying irrigation time series data. Future research can tune the model parameters to improve the accuracy of the prediction results. The method can also be extended to different applications such as gold price prediction, oil price prediction, electricity load prediction, and earthquake prediction to explore its applicability in a broader context.

## Supplemental Information

10.7717/peerj-cs.2112/supp-1 Supplemental Information 1 Source Code

10.7717/peerj-cs.2112/supp-2 Supplemental Information 2 Raw data

## Additional Information and Declarations

### Data Availability

The following information was supplied regarding data availability: The raw data and code are available in the Supplemental Files. The Agrometeorological big data system is available at https://wheata.cn/page/download.html.
